# High Expression of CISD2 in Relation to Adverse Outcome and Abnormal Immune Cell Infiltration in Glioma

**DOI:** 10.1155/2022/8133505

**Published:** 2022-04-21

**Authors:** Fang Zhang, Hua-Bao Cai, Han-Ze Liu, Shen Gao, Bin Wang, Yang-Chun Hu, Hong-Wei Cheng, Jin-Xiu Liu, Yang Gao, Wen-Ming Hong

**Affiliations:** ^1^School of Nursing, Anhui Medical University, Hefei 230032, China; ^2^Department of Neurosurgery, First Affiliated Hospital of Anhui Medical University, Hefei 230032, China; ^3^School of Clinical Medicine, Wan-Nan Medical College, Wuhu 241000, China; ^4^Key Laboratory of Anti-Inflammatory and Immune Medicine, Ministry of Education, Anhui Collaborative Innovation Center of Anti-Inflammatory and Immune Medicine, Institute of Clinical Pharmacology, Anhui Medical University, Hefei 230032, China

## Abstract

Glioma is a serious disease burden globally, with high mortality and recurrence rates. CDGSH iron sulfur domain 2 (CISD2) is an evolutionarily conserved protein that is involved in several cancers. However, its role in the prognosis and immune infiltration in glioma remains unclear. In our research, RNA-seq matrix and clinicopathological relevant data for CISD2 were downloaded from The Cancer Genome Atlas (TCGA) and Genotype-Tissue Expression (GTEx) databases. Human Protein Atlas was used to verify the CISD2 protein level in glioma, and STRING was used to establish relative coexpression gene network. The Kaplan-Meier plotter was adopted to analyze the effect of CISD2 on prognosis. The connection between CISD2 expression and immune infiltration was analyzed using single-sample GSEA (ssGSEA), TIMER, and GEPIA. In contrast to normal tissues, CISD2 expression was significantly higher in glioma tissues, and CISD2 presented a certain diagnostic value in distinguishing glioma tissues from normal tissues. Furthermore, the CISD2 level was correlated with age, histologic grade, histological type, isocitrate dehydrogenase (*IDH*) status, 1p/19q codeletion status, and primary therapy outcome of glioma, while high CISD2 mRNA expression was correlated with grave overall survival. Multivariate analysis demonstrated that CISD2 was an independent risk factor for patients with glioma. Functional enrichment analysis indicated that CISD2 could regulate proliferation, immune reaction, and mitochondrial function. The results from the ssGSEA and TIMER databases confirmed that CISD2 acts a prominent role in immune cell infiltration in the tumor microenvironment, especially in low-grade glioma (LGG). Furthermore, CISD2 expression was observably correlated to M2 polarization in macrophages with glioma progression. This is the first research to investigate the immune role of CISD2 in glioma. CISD2 may be an innovative prognostic biomarker and can act as a potential target for future therapy for glioma.

## 1. Introduction

Glioma is one of the most prevalent subtypes of solid tumors in the central nervous system, and its prevalence has gradually increased year by year [1]. Glioma has high invasiveness and poor prognosis, leading to poor quality of life and short lifespan. The therapy for glioma varies according to cell type, tumor location, tumor size, and grade of malignancy [2]. Currently, the therapeutic options for glioma primarily include surgery, radiotherapy, and chemotherapy. However, patients often have a poor prognosis due to the side effects, such as malignant proliferation of glioma stem cells, leading to tumor recurrence, as well as various functional disorders [3]. Despite considerable progress in the investigation of glioma markers, the lack of explicit markers for glioma grade, subtypes, and prognosis remains a vital obstacle in the management of glioma [4]. Hence, understanding the exact pathogenic mechanisms and latent molecular targets in the tumorigenesis and development of glioma is crucial.

The evolutionarily conserved gene, CDGSH iron sulfur domain 2 (CISD2), is a member of the newly found CDGSH iron sulfur domain (CISD) protein family [5]. CISD2 is enriched in the brain and is also highly expressed in other tissues, such as the thyroid, kidney, and liver [6, 7]. CISD2 is principally localized in the mitochondrial outer membrane and has a vital function in the aging process related to mitochondrial dysfunction [8], human neurodegenerative diseases [9], and various human cancers [10]. Although CISD2 activity is essential for normal development, overexpression of CISD2 has been implicated in several types of human malignancies, including gastric cancer [11], breast cancer [12], and hepatocellular carcinoma [13], indicating that it plays an oncogenic role. Studies have shown that CISD2 expression is increased in glioma and it can induce the proliferation of glioma cells through inhibiting beclin-1-mediated autophagy [14]. However, the knowledge about clinical and prognostic significance of CISD2 in glioma is meager.

A variety of immune cells exist in the tumor microenvironment (TME), and the crosstalk between immune cells and cancer cells has been largely reported [15]. Cancer cells can escape from immune attack through a series of mechanisms, and developing a well-rounded understanding of the role of immune cells in tumorigenesis and applying this knowledge to target therapies in the TME are essential [16]. Previous studies have demonstrated that CISD2 can inhibit inflammatory effects by suppressing the activation of NF-*κ*B in nonstressed microglia [17]. Indeed, in a model of lipopolysaccharide- (LPS-) challenged neural cells, knockdown of CISD2 resulted in a bolstered immune response and significant mitochondrial dysfunction [18]. However, the knowledge of how CISD2 affects immune cell infiltration within the TME in glioma remains deficient.

Our study was to depict the expression profiles of CISD2 and to analyze its latent prognostic role, biological function, and relationship with tumor immune infiltration in patients with glioma. The study outcomes indicated that high CISD2 acted as an indicator of adverse prognosis among patients with glioma and was linked to several carcinogenic and immune-related pathways based on functional enrichment analysis. The findings reveal that CISD2 may be a promising biomarker and a latent therapeutic target for glioma.

## 2. Materials and Methods

### 2.1. Data Collection

The relevant data extracted from The Cancer Genome Atlas (TCGA) and Genotype Tissue Expression (GTEx) are public and do not require the approval of the local ethics committee. The levels of CISD2, clinicopathological details, and general information of glioma were carefully collected. The data consisted of clinical information of patients and RNA-sequencing (RNA-seq) expression of CISD2 in 1157 normal tissues and 689 glioma tissues. To evaluate CISD2 expression, data on glioma tissues were obtained from TCGA database; meanwhile, data on normal tissues were downloaded from TCGA and GTEx databases. The RNA-seq data in transcripts per million reads (TPM) format were converted with log2 for comparison between samples. We generated box plots of the expression difference under the conditions of *P* value cutoff = 0.01 and log2Fold change (FC) cutoff = 1.

The inclusion criteria for patients with glioma in the databases were as follows: (1) patients diagnosed with glioma using immunohistochemical tests; (2) patients who had never received any antitumor therapy before sample collection; and (3) complete case data. In TCGA database, collection of relative data was completed on August 25, 2014. A range of comprehensive therapies were adopted after surgeries, such as radiation, chemotherapy, immunotherapy, and molecular targeted therapy. Patients' disease response to these therapies is evaluated according to Macdonald' criteria (complete response, CR; partial response, PR; stable disease, SD; progressive disease, PD) [19].  Table1.

### 2.2. Human Protein Atlas (HPA) Analysis

The HPA is a database that can provide expression analysis of various proteins in normal and tumor tissues based on immunohistochemical methods [20]. To conduct difference analysis of CISD2 protein expression between normal and glioma tissues, immunohistochemistry images of the cerebral cortex were downloaded from the online HPA database (antibody: HPA015914).

### 2.3. Protein-Protein Interaction (PPI) Comprehensive Analysis

As working molecules of a cell, proteins can perform diverse biological functions through specific interactions with various protein molecules. The online Search Tool for the Retrieval of Interacting Genes/Proteins (STRING) website (https://string-db.org/) was used to map the PPI network between CISD2 and its related differentially expressed genes (DEGs). The website is a well-established source that contains all-round and objective PPI information [21]. First, we selected “Single Protein by Name/Identifier” on the STRING website. Then, we imported “CISD2” into the “Protein Name” input box and selected “Homo sapiens” from the “Organism” box. Finally, a series of main parameters were designed to acquire both known and predicted PPI network information. The association between CISD2 and proteins was represented via a confidence score. The score > 0.4 and *P* < 0.05 were set as the threshold, and a score > 0.7 indicated high correlation.

### 2.4. Coexpression Network Analysis

To identify the target genes that were coexpressed with CISD2 in glioma, we performed a relative analysis using the LinkedOmics database (http://www.linkedomics.org/login.php), which can be used for the analysis of 32 TCGA cancer-associated multidimensional datasets [22]. The genes were considered interesting DEGs when they were up to a standard of adj. *P* < 0.05 and |logFC| > 1.0. Then, a volcano plot was adopted to exhibit the enrichment of CISD2-related DEGs. Meanwhile, to obtain genes coexpressed with CISD2 in glioma, we set the following filtering criteria: |correlation coefficient| > 0.3 and *P* < 0.05. The correlation values were sorted in descending or ascending order, and we separately obtained the top 50 positive or negative coexpressed genes, which were displayed in the form of a heat map.

### 2.5. Gene Ontology (GO) and Kyoto Encyclopedia of Genes and Genomes (KEGG) Enrichment Analyses

We used the 50 most positive coexpression genes with CISD2 in glioma for GO and KEGG analyses via the “clusterprofiler” R package. These GO terms consisted of biological process (BP), cellular component (CC), and molecular function (MF). And the top five significant pathways were sorted (*P* < 0.05).

### 2.6. Gene Set Enrichment Analysis (GSEA)

GSEA is an algorithmic technique that indicates whether a set of a priori defined genes has statistically significant differences in expression under two biological states [23]. First, GSEA provided an organized list of all genes based on their correlation with CISD2 expression. Next, GSEA was set out to reveal the prominent survival difference detected between high- and low-CISD2 expression groups. The standards of nominal *P* value < 0.05, normalized enrichment score > 1.7, and false discovery rate < 0.25 were adopted to select the pathways that were markedly enriched.

### 2.7. Analysis of Immune Infiltrates by Single-Sample GSEA (ssGSEA)

The ssGSEA concentrating on gene sets can show general information on chromosomal location, biological function, or regulation [24]. In this research, the ssGSEA algorithm was used to filtrate the relative quantity of immune cells infiltrating in the TME of glioma. The marker gene sets for various infiltrating immune cell types in the TME of glioma were gained and merged according to the research of Bindea et al. [25]. We investigated the infiltrating immune cells, which included innate immune cells (such as neutrophils, eosinophils, mast cells, and macrophages) and adaptive immune cells (Treg cells, T cells, B cells, T helper, and cytotoxic cells). The relationship between CISD2 and infiltrating immune cells was determined by ssGSEA via the R package “GSVA.”

### 2.8. Analysis of Immune Infiltrates by TIMER and GEPIA

As a web server, the tumor immune estimation resource (TIMER) is commonly used for rounded and synthetical analysis of immune infiltrates in many cancers (http://cistrome.org/TIMER/) [26]. The “Gene” module of TIMER can provide data, including the purity-corrected partial Spearman's rho value and statistical significance, on the relationship between CISD2 expression and abundance of infiltrating immune cells in glioma. Meanwhile, the “Correlation” module supplied the data on CISD2 and immune cell markers in glioma, which included the *P* value and partial correlation based on purity-adjusted Spearman's rank correlation test. Additionally, the “Survival” module was used to explore the clinical relevance of the tumor immune subsets with CISD2 expression. Survival differences were visualized via TIMER, which can draw the Kaplan-Meier plots for immune infiltrates and CISD2 expression. We set the expression thresholds for splitting the high and low expression groups with cutoff-high (50%) and cutoff-low (50%) values. Then, we compared the survival curves of the two groups, which are shown in each plot via the *P* value of the log-rank test. Gene Expression Profiling Interactive Analysis (GEPIA) focuses on the extensive analyses of single-cell RNA-seq datasets [27]. The correlation between CISD2 and various immune markers in glioma was investigated by R package *corrplot*. The *x*-axis presents the CISD2 expression level, and the corresponding *y*-axis was plotted with other known immune genes in GEPIA. The TIMER was used to validate a set of genes that had a remarkable correlation with CISD2 in the GEPIA web.

### 2.9. Statistical Analysis

Data processing and analysis were performed using R (v.3.6.3). The discrete differences in CISD2 mRNA expression among types were visualized through box plots by ggplot2. We sought a correlation between CISD2 expression and clinical characteristics. The Mann-Whitney U or Kruskal-Wallis tests were used to compare the gene expression profiles across samples. The diagnostic value of CISD2 in glioma was evaluated based on the specificity and sensitivity generated by the receiver operating characteristic (ROC) curve. The prognostic value of CISD2 was judged by the “survival” package. The combination of the Kaplan-Meier method and stratified log-rank test was used to analyze the prognostic value of CISD2. In order to compare the effect of CISD2 on survival along with other clinical characteristics, the univariate and multivariate analyses based on the Cox regression model were used. The GEPIA data were revealed as hazard ratio (HR) *P* values or Cox *P* values from the log-rank test. In the TIMER, Spearman's correlation coefficient was adopted to estimate the correlation of gene expression, and *P* < 0.05 was considered significant.

## 3. Results

### 3.1. Upregulated CISD2 Expression Predicts Dismal Outcomes in Patients with Glioma

**Figure 1:**
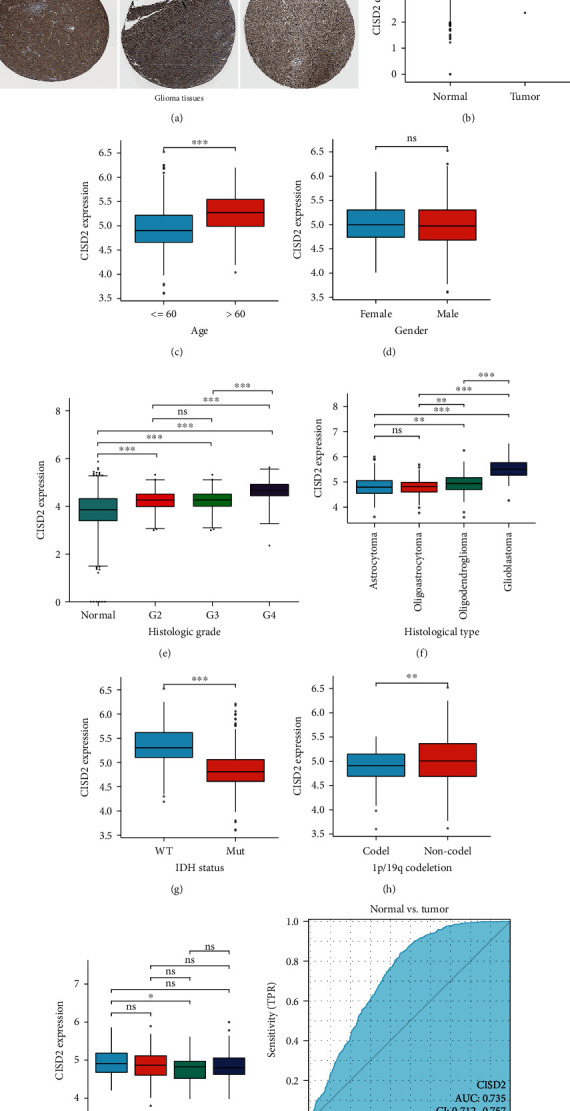
Upregulated CISD2 expression predicts dismal outcomes in patients with glioma. (a) The protein levels of CISD2 in glioma from the HPA database. Brown (black) arrows reveal CISD2-positive staining. (b) CISD2 mRNA expression in glioma and normal tissues. (c–i) CISD2 mRNA expression in various subgroups, including (c) age, (d) sex, (e) histologic grade, (f) histological type, (g) *IDH* status, (h) 1p/19q codeletion status, and (i) primary therapy outcome. (j) Receiver operating characteristic curve analysis of tissue CISD2 expression to detect patients with glioma. ∗*P* < 0.05, ∗∗*P* < 0.01, and ∗∗∗*P* < 0.001.

We first examined the transcription levels of CISD2 using the data from TCGA. In comparison with normal tissues, CISD2 mRNA expression was dramatically increased in glioma tissues (Figure 1(b)). Furthermore, the immunohistochemistry results from HPA revealed that CISD2 showed higher expression in glioma specimens than in normal specimens (Figure 1(a)). Additionally, CISD2 mRNA expression was checked in different subgroups based on age, sex, histologic grade, histological type, *IDH* status, 1p/19q codeletion status, and primary therapy outcome. Specifically, CISD2 mRNA expression was observably increased in patients older than 60 years (Figure 1(c)). Moreover, the expression of CISD2 increased gradually from G2 to G4, and CISD2 in G4 showed significantly higher expression than in G2 or G3 (Figure 1(e)). However, no significant relationship was found between the expression of CISD2 mRNA and sex (Figure 1(d)). CISD2 mRNA expression levels were remarkedly increased with the malignant grade according to histological type (Figure 1(f)). Several reports have shown that *IDH* mutation and 1p/19q codeletion status have prognostic importance in glioma [28]. We observed significantly lower expression levels of CISD2 mRNA in *IDH* mutation tumors compared to *IDH*-wild-type tumors (Figure 1(g)), and CISD2 mRNA expression was remarkably decreased in 1p/19q codeleted tumors compared to 1p/19q noncodeleted tumors (Figure 1(h)). In terms of the primary therapy outcome, we found CISD2 markedly upregulated in the PD group than in the PR group (Figure 1(i)). Through ROC curve analysis, the area under the curve (AUC) shown in Figure 1(j) was 0.735, revealing that CISD2 has auxiliary diagnostic significance in distinguishing glioma tissues from normal tissues. Additionally, the sensitivity was 83.9% and the specificity was 55.1% when the cutoff value was 3.95.

### 3.2. Correlation between CISD2 Expression and Clinical Features of Patients with Glioma

In order to develop in-depth knowledge of the clinical significance of CISD2, we analyzed the correlation between it and various clinical features in glioma. The results showed that CISD2 was correlated with age (*P* < 0.001), histologic grade (*P* < 0.001), *IDH* status (*P* < 0.001), 1p/19q codeletion status (*P* = 0.013), histological type (*P* < 0.001), and primary therapy outcome (*P* = 0.014) (Table 1). These results indicated that CISD2 is positively associated with poor outcomes in glioma.

### 3.3. Prognostic Value of CISD2 Expression in Patients with Glioma

**Figure 2:**
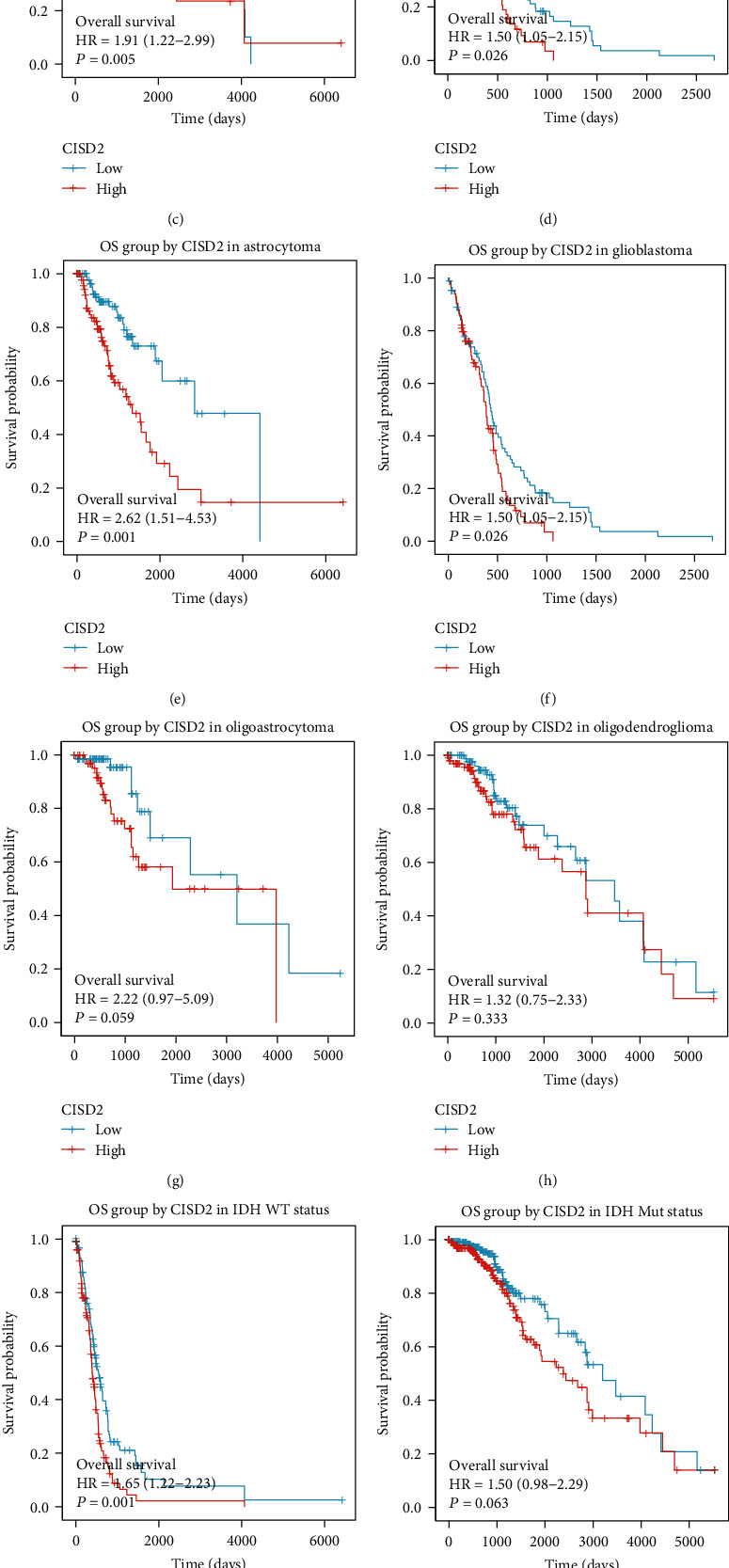
Prognostic value of CISD2 expression in patients with glioma. (a) OS of patients with glioma in low and high CISD2 expression. (b–l) OS of patients with glioma based on low or high CISD2 expression among various subgroups, including (b) G2, (c) G3, (d) G4, (e) astrocytoma, (f) glioblastoma, (g) oligoastrocytoma, (h) oligodendroglioma, (i) *IDH*-wild-type status, (j) *IDH* mutation status, (k) 1p/19q codeletion status, and (l) 1p/19q noncodeletion status. OS: overall survival.

Prognostic value of CISD2 expression in glioma was identified by the Kaplan-Meier survival curve analysis. As shown in Figure 2(a), patients with glioma with high CISD2 expression showed remarkably shorter overall survival (OS) than patients with low CISD2 expression. Besides, subgroup analysis revealed that patients with high CISD2 expression showed poorer OS in cases with G3 (*P* = 0.005), G4 (*P* = 0.026), astrocytoma (*P* = 0.001), and glioblastoma (*P* = 0.026) (Figures 2(c)–2(f)). Moreover, the OS was significantly shorter in patients with glioma with higher CISD2 expression than in those with lower CISD2 expression in the *IDH*-wild-type group and 1p/19q noncodeleted group (Figure 2(i) and 2(l)). This suggests that CISD2 expression may influence the prognosis in patients with glioma.

### 3.4. Cox Univariate and Multivariate Analyses of Risk Factors for OS in Patients with Glioma

As CISD2 expression was linked with OS in patients with glioma, it was of great clinical significance to identify the underlying mechanism. Therefore, the relationship between CISD2, clinical characteristics, and OS was probed. Univariate Cox analysis revealed poor OS-related factors, including age > 60 years, high histologic grade, *IDH*-wild-type, 1p/19q noncodeletion status, and high CISD2 expression in patients with glioma. Multivariate Cox analysis showed that high CISD2 expression was an independent risk factor for poor OS in patients with glioma (Table 2).

### 3.5. CISD2 Coexpression Networks in Glioma

**Figure 3:**
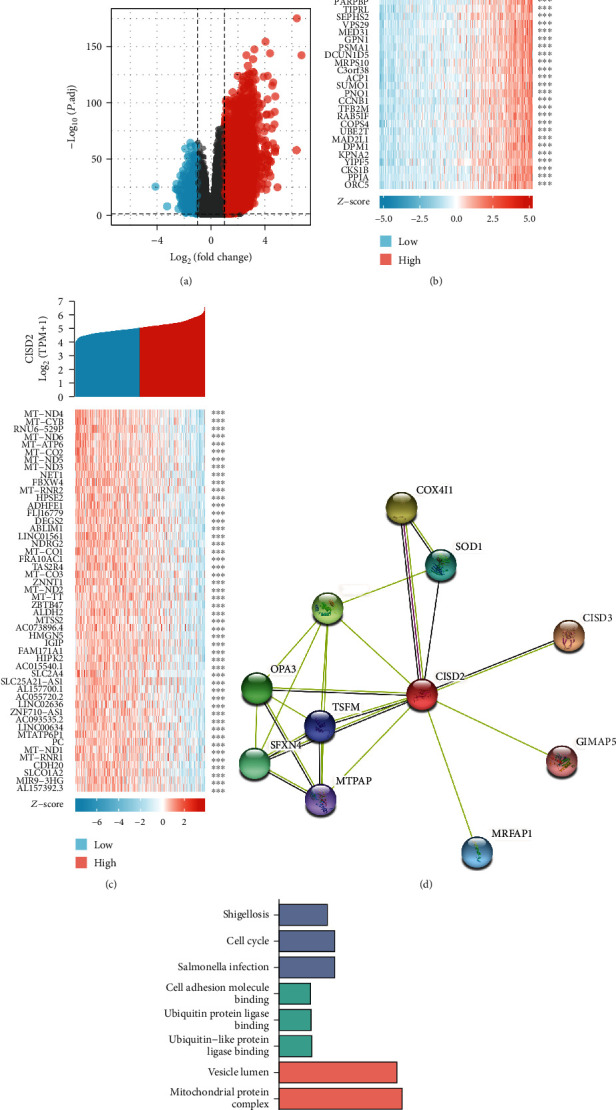
CISD2 coexpression genes in glioma. (a) Correlation between CISD2 and genes differentially expressed in glioma. (b) Heat map of the 50 significant genes positively correlated with CISD2 in glioma. (c) Heat map of the 50 most significant genes negatively correlated with CISD2 in glioma. (d) CISD2-interaction proteins in glioma tissues. Annotation of CISD2-interaction proteins and their coexpression scores. (e) Significantly enriched GO annotations (CC: cellular component; BP: biological process; MF: molecular function) and KEGG pathways of CISD2 and 50 most positive coexpression genes in glioma.

To increase the comprehension of the biological meaning of CISD2 in glioma, we used the function module of LinkedOmics to examine CISD2 coexpression modes in the glioma cohort.  Figure 3(a) shows that a set of highly DEGs were associated with CISD2 based on Pearson's correlation. To facilitate comparison, we used red and green dots to label genes that were positively and inversely correlated with CISD2, respectively. Besides, the top 50 significant coexpression genes positively and negatively correlated with CISD2 are shown in the heat maps (Figures 3(b) and 3(c) and Table S1, 2).

The classification of PPI has gained attention because it is an essential element of the intricate and complex network of cellular interactions [29]. The identification of PPI networks is the foundation of understanding functional genomics. Hence, we constructed a dynamic PPI network of CISD2-related DEGs to evaluate their cross-action in glioma through STRING (Figure 3(d) and Table 3). The top 10 genes included CISD3, COX4I1, WFS1, OPA3, SFXN4, SOD1, MRFAP1, TSFM, MTPAP, and GIMAP5. Recent evidence suggests that CISD3 is necessary for tumor cell proliferation by inhibiting cell death [30]. Moreover, increased expression of COX4I1 is correlated with shorter progression-free and OS in patients with glioblastoma multiforme (GBM) [31].

### 3.6. Enrichment Analyses of CISD2-Related Genes in Glioma

Enrichment analyses of GO and KEGG pathways were performed based on the 50 most positive coexpressed CISD2-related genes. We detected enrichment in GO terms of a few biological processes, such as immune response, neutrophil activation, and neutrophil degranulation. In terms of cellular composition, vesicle lumen, mitochondrial protein complex, and mitochondrial inner membrane were the top three significantly enriched GO terms. Moreover, CISD2-related genes were involved in the regulation of cell adhesion molecule binding, ubiquitin protein ligase binding, and ubiquitin-like protein ligase binding. KEGG analysis demonstrated enrichment in the pathways associated with shigellosis, cell cycle, and salmonella infection (Figure 3(e) and Table S3).

### 3.7. GSEA Investigation of CISD2-Related Pathways

**Figure 4:**
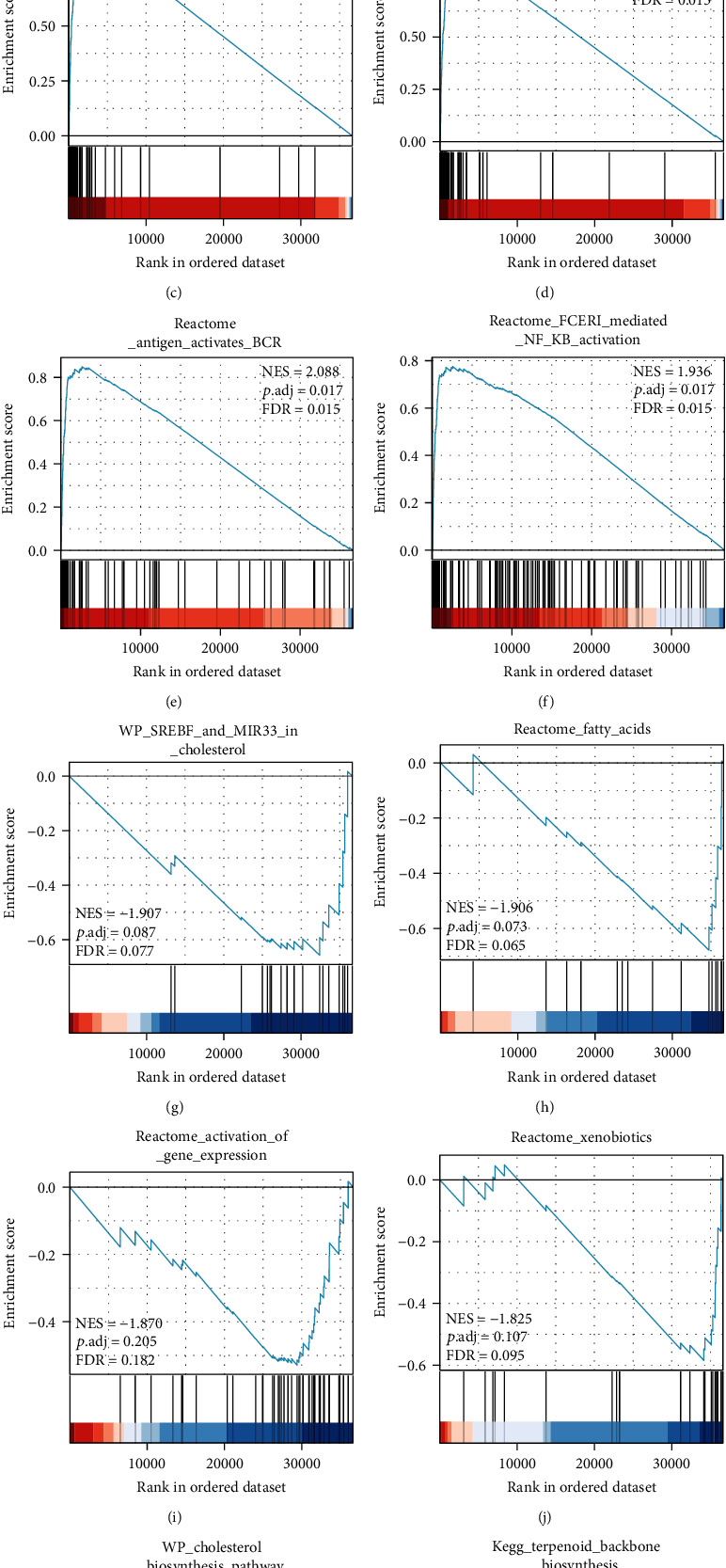
GSEA data exhibited the most relevant gene sets. (a–f) Upregulated gene sets in the high CISD2 expression group. (g–l) Downregulated gene sets in the high CISD2 expression group. NES: normalized enrichment score; *P* adj: adjusted *P* value; FDR: false discovery rate.

Next, we sought to identify the biological pathways and processes correlated with CISD2. We extracted the most prominently enriched signaling pathways based on the absolute value of normalized enrichment score. Pathways related to HEME scavenging in plasma, FCGR activation, NF-*κ*B activation, and B cell activation were significantly displayed by a high CISD2 expression phenotype, while pathways related to cholesterol and lipid homeostasis and fatty acid metabolism showed a low CISD2 expression phenotype (Figures 4(a)–4(l) and Table 4).

### 3.8. CISD2 Expression Is Related to Tumor-Infiltrating Immune Cells in Glioma with ssGSEA

**Figure 5:**
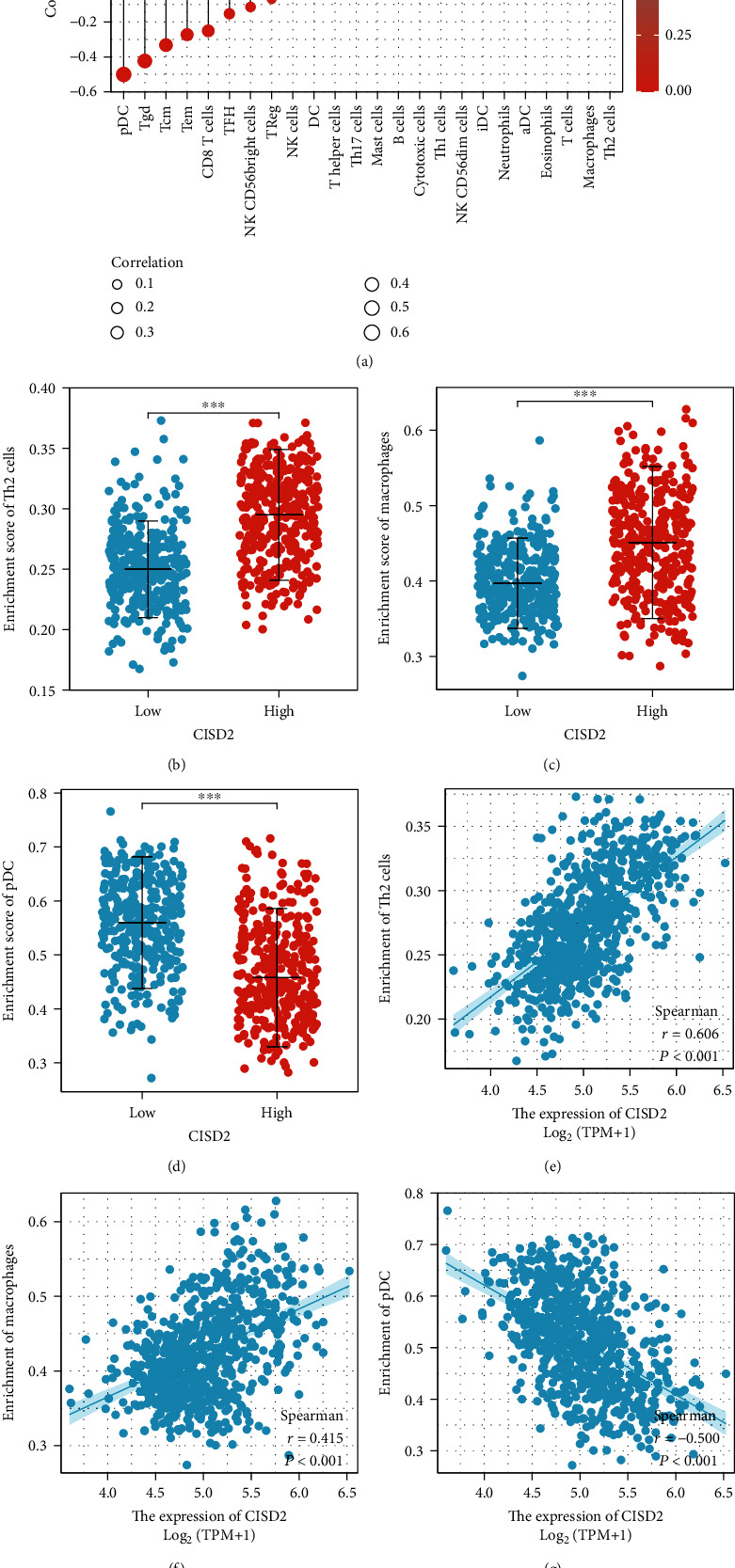
CISD2 expression is related to tumor-infiltrating immune cells in glioma. (a) Correlation between various immune cells and CISD2 expression with ssGSEA. (b–g) The diversity of the Th2 cell, macrophage, and pDC infiltration level between CISD2-high and CISD2-low groups is shown using scatter plots and correlation diagrams.

The results of GO, KEGG, and GSEA analyses indicated that CISD2 was linked to immune response. As a hallmark of tumors, tumor-infiltrating immune cells have an important impact on patient's prognosis [32]. Therefore, we tried to further investigate the infiltration of immune cells in glioma by ssGSEA. CISD2 positively affected the abundance of immunocytes (such as helper T2 [Th2] cells, macrophages, and T cells) and negatively affected the abundance of immunocytes (plasmacytoid dendritic cells [pDCs], gamma delta T [Tgd] cells, and central memory T cell [Tcm]) (Figures 5(a)–5(g), *P* < 0.001).

### 3.9. CISD2 Expression Is Correlated with Immune Infiltration Level and Cumulative Survival in Glioma with TIMER

**Figure 6:**
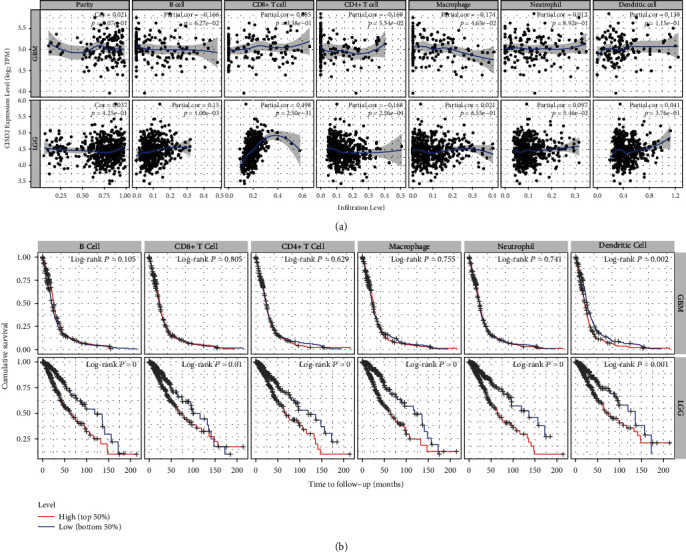
CISD2 expression is correlated with immune infiltration and cumulative survival in glioma with TIMER. (a) CISD2 expression shows a notably inverse correlation with macrophages and no correlation with B cells, CD8^+^ T cells, CD4^+^ T cells, neutrophils, and DCs in GBM. Meanwhile, CISD2 expression is notably positively correlated with B cells, CD8^+^ T cells, and neutrophils and negatively correlated with CD4^+^ T cells in LGG. (b) The Kaplan-Meier curves analyses of immune cell infiltration and CISD2 expression in GBM and LGG. GBM: glioblastoma multiforme; LGG: low-grade glioma.

Recent evidences have shown that glioma purity takes an important role under genomic, clinical, and biological conditions [33, 34]. To acquire a precise prediction, it is essential to integrate glioma purity into the relative evaluation system. Next, we investigated the effects of CISD2 on immune infiltration levels in patients with glioma using TIMER. The CISD2 expression level in both GBM and low-grade glioma (LGG) had no significant relationship with tumor purity, indicating that it is uniformly expressed in the TME. Besides, a weak and negative correlation exists between the CISD2 expression and expression of macrophages (*r* = −0.174, *P* = 4.65e − 02) in GBM. For LGG, CISD2 expression was weakly positively correlated with the expression of B cells (*r* = 0.15, *P* = 1.00e − 03) and neutrophils (*r* = 0.097, *P* = 3.46e − 02) and weakly negatively correlated with CD4^+^ T cells (*r* = −0.168, *P* = 2.26e − 04). Interestingly, the results showed that CISD2 was moderately and positively correlated with CD8^+^ T cells (*r* = 0.498, *P* = 2.50e − 31) in LGG but was not significantly linked to CD8^+^ T cells in GBM, indicating that there was a decreased tendency of a relationship between CISD2 and CD8^+^ T cells during the progression of glioma (Figure 6(a)). Additionally, the cumulative survival rate was significantly related to CISD2 expression and the infiltration of B cells, CD8^+^ T cells, CD4^+^ T cells, macrophages, neutrophils, and DCs in patients with LGG over time (Figure 6(b)). These data suggest that CISD2 plays a significant role in immune cell infiltration in LGG.

### 3.10. Correlations between CISD2 Expression and Immune Markers in Glioma

To further investigate the latent effect of CISD2 on infiltrating immune cells, we probed the relationships between CISD2 and various immune cell markers. According to the adjustments for tumor purity, CISD2 expression demonstrated a significant correlation with most of the gene markers of functional T cells (CD8^+^ T, Th1, Th2, Treg, and exhausted T cells), B cells, and neutrophils in LGG. However, only a few makers for B cells, CD8^+^ T cells, neutrophils, Th1, Th2, and Treg were remarkably linked to CISD2 expression in GBM (Table 5). These data suggest that CISD2 has a significant role in immune cell infiltration in glioma, especially in LGG. These data might explain the prognostic difference in CISD2 in LGG and GBM to some extent.

### 3.11. Correlation between CISD2 Expression and Macrophage Polarization in Glioma

**Figure 7:**
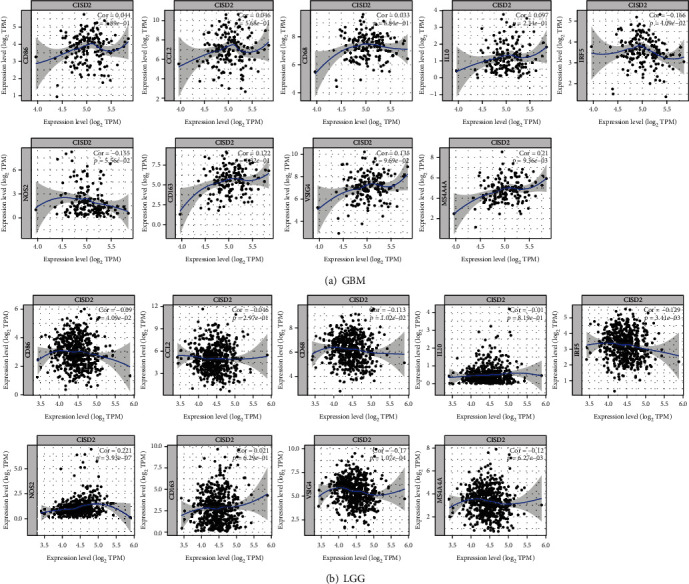
CISD2 expression is related to macrophage polarization in GBM and LGG. Markers include CD86 (monocytes); CD68, CCL2, and IL10 (TAM); IRF5 and NOS2 (M1 macrophage); and CD163, VSIG4, and MS4A4A (M2 macrophage). GBM: glioblastoma multiforme; LGG: low-grade glioma; TAM: tumor-associated macrophage.

Studies have shown that macrophage polarization from the classically activated macrophage (M1) phenotype to the alternatively activated (M2) phenotype is correlated with tumor development [35]. The results shown in Figure 7 demonstrate various relationships between CISD2 levels and macrophage markers in GBM and LGG. Interestingly, CISD2 expression in GBM and LGG showed different relationships with markers of monocytes, TAM, M1, and M2. CISD2 in GBM had significant positive correlation with M2 marker (MS4A4A), while CISD2 in LGG had significant negative correlations with M2 markers (VSIG4 and MS4A4A). Moreover, CISD2 in GBM had significant negative correlations with M1 marker (IRF5), while CISD2 in LGG had significant positive correlations with M1 marker (NOS2) (Figure 7). Collectively, these data indicate that CISD2 may induce M2 polarization in macrophages with glioma progression.

## 4. Discussion

Glioma has rapid disease progression and can cause various clinical symptoms due to its size and location [36]. The pathogenesis of glioma is complicated, which is consistent with aberrant gene expression that affects cell growth, invasiveness, and angiogenesis [37, 38]. Generally, low-grade tumors grow more slowly and have a more favorable prognosis than high-grade tumors. However, the pathological grade is insufficient to predict the prognosis of patients with glioma [2]. Therefore, it is necessary to find promising biomarkers to help understand the biological characteristics of glioma and predict its clinical prognosis. Here, we found that CISD2 is overexpressed in glioma and plays a role in its diagnosis. And increased CISD2 expression was signally relevant to advanced clinical pathological parameters, shorter survival time, and poor prognosis. CISD2 is overexpressed in many glioma samples and is worthy of further verification in the clinic as a possible diagnostic and prognostic marker. Our group first comprehensively probed the role of CISD2 in the TME in glioma. These results indicate that CISD2 expression is differently related to tumor-infiltrating immune cells in the TME in LGG and GBM, which may partly contribute to their prognostic difference.

CISD2 was originally regarded as a prolongevity gene related to the function of mitochondria and the endoplasmic reticulum, and it is known to be involved in many biological processes [39]. As tumor cells exhibit the ability to grow indefinitely and can survive for a long time, the role of CISD2 in tumors has attracted much interest. CISD2 may activate the WNT/*β*-catenin pathway and promote epithelial-mesenchymal transformation in pancreatic cancer [40]. In hepatocellular carcinoma, the expression of CISD2 is associated with advanced clinicopathological characteristics, such as tumor size, number of tumors, surgical margin, recurrence, and poor prognosis [13]. Shao et al. [41] revealed that CISD2 could be developed as a chemotherapeutic target in human breast cancer owing to its effect on cell proliferation. And Sun et al. [14] reported that CISD2 was significantly increased in glioma tissues and could promote cell proliferation by inhibiting beclin-1-mediated autophagy. Thus, it is reasonable to speculate that CISD2 may play a prominent role in glioma. Our systematic bioinformatics analysis demonstrated that increased expression of CISD2 in glioma was abnormally associated with poor clinicopathological parameters (high histologic grade and malignant histological type). The presence of *IDH* mutations and 1p/19q codeletions is related to a more favorable clinical outcome, with increased sensitivity to some chemotherapy drugs, such as procarbazine, lomustine, and vincristine [42]. Our data showed that high level of CISD2 expression was notably correlated with *IDH*-wild-type tumors and 1p/19q noncodeleted tumors. Specifically, we found that CISD2 expression was not only lower in oligodendroglioma than in GBM but was also lower in the 1p/19q codeleted tumors compared to the 1p/19q noncodeleted tumors. This is in line with the previous research which has demonstrated that patients with 1p/19q codeletion have a more favorable prognosis than those with no codeletion or 1p or 19q codeletion alone in oligodendroglioma [43]. Masoudi et al. [44] reported that patients diagnosed with PD or SD have poorer survival times than those diagnosed with SD in high-grade glioma. We found a significantly higher expression of CISD2 in patients achieved with PD than with PR, indicating that CISD2 may take part in pathologic progression of glioma. Collectively, these results further highlighted that increased CISD2 expression was linked to adverse outcomes in glioma.

The main treatment of glioma is surgery supplemented by radiotherapy and chemotherapy as well as other combined therapies. However, only patients diagnosed at an early stage can achieve good therapeutic effects, and the outcomes of patients diagnosed at advanced stages are often negative [45]. The AUC of ROC analysis was 0.735, indicating that CISD2 has certain diagnostic value in screening glioma tissues from normal tissues. High CISD2 expression was significantly correlated with poor prognosis in different tumors. In gastric cancer, CISD2 is significantly upregulated and is markedly associated with clinical stage, venous invasion, TNM classification, and lymphatic invasion [46]. Yang et al. [40] found that CISD2 expression was abnormally upregulated in human laryngeal squamous cell carcinoma tissues and was remarkably correlated with T stage, lymphatic invasion, clinical stage, and progress of the disease. Upregulation of CISD2 is also found in early-stage cervical cancer and is linked to adverse prognosis [12]. In human pancreatic cancer, strong CISD2 expression showed a positive relationship with advanced vascular invasion, distant metastasis, clinical stage, T-stage, and larger tumor diameter [47]. Consistently, we found that high CISD2 expression was markedly associated with poorer clinical features and OS, and multivariate regression analysis revealed it was an independent risk factor for poor OS.

To investigate the functions of CISD2 in glioma in-depth, we constructed functional networks based on the coexpressed genes. The results of GO and KEGG pathway analyses indicated that these CISD2 coexpressed genes were involved in various biological processes, including immune response and mitochondrial function regulation and signaling pathways related to cell cycle and microbism. Through GSEA, overexpression of CISD2 was found to be correlated with various signal transduction processes, such as the NF-*κ*B pathway, B cell receptor pathway, and complement cascade, and other immunoregulatory interaction pathways. These signal transduction processes and biological pathways are linked to glioma carcinogenesis [48]. However, *in vivo* and *in vitro* experiments are needed to verify these possible pathways and processes regulated by CISD2 in glioma.

The TME includes a repertoire of cell clusters (such as tumor cells, immune cells, and fibroblasts) and plays a diverse role in cancer biology [49]. Previous research suggested that the main difficulty in treating glioma is the therapeutic resistance owing to its complex metabolic characteristics and highly immunosuppressive microenvironment [50]. However, the relationship between CISD2 and immune infiltration in glioma remains unclear. Our study indicated that the expression of CISD2 was positively correlated with Th2 cells, macrophages, and T cells and was negatively correlated with pDCs, Tgd, and Tcm. It has been proven that the infiltration of lymphocytes is rare in glioma because it is difficult for lymphocytes to transport from the periphery to the TME due to the existence of the blood-brain barrier [51]. Patients diagnosed with malignant glioma usually present with immune deficiency, especially T cell dysfunction [52]. Consistently, the analysis of the relationship between CISD2 and immune cell markers showed that the vast majority of correlation values were very low. But the statistical results showed that CISD2 expression was significantly correlated with several types of tumor-infiltrating immune cells in LGG, including B cells, CD8^+^ T cells, neutrophils, and CD4^+^ T cells in TIMER. However, CISD2 expression was only markedly linked to macrophages in GBM. We found that CISD2 had a notable and positive relationship with B cells in LGG, although the reverse was observed in GBM. One possible reason for this finding is that although there are various immune cells in the TME, the immune responses are highly suppressed in GBM [53]. Recent research has indicated that tumor-infiltrating B cells play a paradoxical role in some tumors. B cells can lead to favorable outcomes through interacting with T cells and other immune cells but can also inhibit the progression of immune responses under other conditions [54, 55]. It is well known that patients newly diagnosed with glioma who have more CD8^+^ T cell infiltration have a better prognosis than those with less CD8^+^ T cell infiltration [56]. Consistently, the ssGSEA results showed that CISD2 had a notable and negative relationship with the abundance of CD8^+^ T cells in glioma. However, the results of TIMER showed that CISD2 significantly and positively correlated with CD8^+^ T cells in LGG, but this positive relationship was not statistically significant in GBM. In other words, these results were inconsistent with the data obtained in ssGSEA, which may be due to the different computational algorithms in data manipulation. The relative analysis was performed based on a nonparametric and unsupervised algorithm in ssGSEA, while it was conducted based on deconvolution methods in TIMER [57]. Besides, the sample volumes were different in the analysis (703 in ssGSEA and 10897 in TIMER) [58]. Another probable reason for this discrepancy may be that different immune characteristics were formed at different stages of glioma development. A recent study suggested that there were more CD8^+^ T lymphocytes in glioma tissues than in normal tissues, and they were also higher in GBM than in LGG, indicating that infiltration levels of CD8^+^ T cells changed with the tumor progression [59]. Furthermore, they also suggested that CD8^+^ T cell expression was positively related to patients' prognosis. Consistently, we found that the correlation value of a positive relationship between CISD2 expression and CD8^+^ T cells was significantly decreased with glioma progression in TIMER, indicating that CISD2 might exert an inhibitory effect on CD8^+^ T cells with tumor progression. Further experiments, single-cell RNA-seq, and clinical validation using large numbers of samples are needed to confirm this relationship.

The relationships between gene markers of different immune cells and CISD2 expression highlighted the significant meaning of CISD2 in modulating the TME of glioma, especially in LGG. In our study, the data showed that CISD2 expression was observably related to some marker sets. CISD2 expression was observably related to CD8A of CD8^+^ T cells, IRF5 of M1, MS4A4A of M2, CCR7 of neutrophils, STAT1 of Th1, STAT5A of Th2, and STAT5B of Treg in LGG and GBM. According to the adjustments for tumor purity, CISD2 expression demonstrated a significant correlation with 8 out of 51 immune cell markers in GBM and 30 out of 51 immune cell markers in LGG. These data indicate that CISD2 plays a specific role in immune cell infiltration in glioma, especially in LGG. We observed that CISD2 was significantly related to most of the markers of Th1, Th2, and Treg cells in LGG, while it was related to rare markers of these cells in GBM, indicating that CISD2 dynamically modulates T lymphocyte immunity with glioma progression. The macrophages ubiquitously exist in most organs and take part in the progression of various diseases, such as atherosclerosis [60], diabetes [61], and cancer [62]. Cumulative data demonstrated that the polarization of macrophages from the antitumor M1 phenotype to protumor M2 phenotype was related to tumor progression and that a higher density of M2 TAMs is tightly linked to an adverse prognosis in cancer [62, 63]. As a signature of M2-polarized macrophages, MS4A4A is believed to be unfavorable and is involved in several cancers, including gastric [64], melanoma [65], and ovarian cancer [66]. A previous study suggested that the suppression of MS4A4A^+^ macrophages is associated with relatively good outcomes of some pathological conditions [67]. The results of our investigation demonstrated that CISD2 expression was significantly and negatively correlated with MS4A4A in LGG, whereas it had a significantly positive correlation with MS4A4A in GBM, suggesting the potential administrative role of CISD2 in macrophage polarization. As a marker used to define M1 macrophages, the higher abundance of NOS2 in tumor islets is often related to better prognosis. We found that CISD2 significantly and positively correlated with NOS2 in LGG, whereas it had a negative correlation with NOS2 in GBM. Collectively, these bioinformatics data suggest that CISD2 could modulate macrophage polarization from M1 to M2 with tumor progression, providing a direction for further research.

The study has several limitations. First, we only performed bioinformatics analysis using several major databases. However, our study focuses on the clinical significance without exploring the molecular mechanism of CISD2 in glioma. Second, it is better to set up a prognostic predictive model by integrating CISD2 expression and all the variables using machine learning. Third, there was systematic bias of the analysis of immune cell infiltration across databases. In the future, analysis by high resolution, prospective studies, in *vivo*/*in vitro* experiments, and clinical validation using a large number of samples should be performed to explore the biological significance of CISD2.

## 5. Conclusion

In conclusion, our study demonstrates that CISD2 expression levels can be used to diagnose and evaluate the prognosis of patients with glioma. CISD2 may be involved in the TME through regulating tumor-infiltrating immune cells in glioma. CISD2 may be an innovative prognostic biomarker and can act as a potential target for future therapy of glioma.

## Figures and Tables

**Table 1:** Relationship between CISD2 mRNA expression and clinical features in glioma.

Characteristics	Variable	Low expression of CISD2	High expression of CISD2	*χ* ^2^	*P*
*n*		348	348		

Age, *n* (%)	≤60	314 (45.1%)	239 (34.3%)	48.2	<0.001
	>60	34 (4.9%)	109 (15.7%)		

Gender, *n* (%)	Female	145 (20.8%)	153 (22%)	0.29	0.592
	Male	203 (29.2%)	195 (28%)		

Histologic grade, *n* (%)	G2	155 (24.4%)	69 (10.9%)	180.11	<0.001
	G3	137 (21.6%)	106 (16.7%)		
	G4	6 (0.9%)	162 (25.5%)		

*IDH* status, *n* (%)	WT	43 (6.3%)	203 (29.6%)	164.6	<0.001
	Mut	303 (44.2%)	137 (20%)		

1p/19q codeletion, *n* (%)	Codel	101 (14.7%)	70 (10.2%)	6.21	0.013
	Noncodel	247 (35.8%)	271 (39.3%)		

Histological type, *n* (%)	Astrocytoma	135 (19.4%)	60 (8.6%)	204.2	<0.001
	Glioblastoma	6 (0.9%)	162 (23.3%)		
	Oligoastrocytoma	98 (14.1%)	36 (5.2%)		
	Oligodendroglioma	109 (15.7%)	90 (12.9%)		

Primary therapy outcome, *n* (%)	PD	62 (13.4%)	50 (10.8%)	10.54	0.014
	SD	90 (19.5%)	57 (12.3%)		
	PR	49 (10.6%)	15 (3.2%)		
	CR	97 (21%)	42 (9.1%)		

CISD2: CDGSH iron sulfur domain 2; *IDH*: isocitrate dehydrogenase; PD: progressive disease; SD: stable disease; PR: partial response; CR: complete response.

**Table 2:** Correlations between overall survival and mRNA expression of CISD2 analyzed by univariate and multivariate Cox regression.

Characteristics	Total (*N*)	Univariate analysis		Multivariate analysis	
		Hazard ratio (95% CI)	*P* value	Hazard ratio (95% CI)	*P* value
Age (>60 vs. ≤60)	695	4.668 (3.598–6.056)	<0.001	1.621 (1.189–2.209)	0.002
Gender (male vs. female)	695	1.262 (0.988–1.610)	0.062	—	
Histologic grade (G3 vs. G2)	466	2.999 (2.007–4.480)	<0.001	1.929 (1.256–2.964)	0.003
Histologic grade (G4 vs. G2)	391	18.615 (12.460–27.812)	<0.001	4.172 (2.463–-7.067)	<0.001
*IDH* status (WT vs. Mut)	685	8.551 (6.558–11.150)	<0.001	3.907 (2.701–5.652)	<0.001
1p/19q codeletion (noncodel vs. codel)	688	4.428 (2.885–6.799)	<0.001	1.763 (1.063–2.923)	0.028
CISD2 (high vs. low)	695	4.253 (3.231–5.596)	<0.001	2.053 (1.442–2.924)	<0.001

CISD2: CDGSH iron sulfur domain 2; CI: confidence interval; *IDH*: isocitrate dehydrogenase.

**Table 3:** 

Gene symbol	Annotation	Score
CISD3	CDGSH iron sulfur domain-containing protein 3	0.883
COX4I1	Cytochrome c oxidase subunit 4 isoform 1	0.866
WFS1	Wolframin	0.863
OPA3	Optic atrophy 3	0.735
SFXN4	Sideroflexin-4	0.716
SOD1	Superoxide dismutase	0.692
MRFAP1	Morf4 family associated protein 1	0.691
TSFM	Elongation factor Ts	0.666
MTPAP	Poly(A) RNA polymerase	0.664
GIMAP5	GTPase IMAP family member 5	0.655

**Table 4:** GSEA pathways up- and downregulated due to expression of CISD2.

Gene set name	NES	NOM *P* value	*P* adjust	FDR *q* value
REACTOME_SCAVENGING_OF_HEME_FROM_PLASMA	2.168	0.001	0.017	0.015
REACTOME_CD22_MEDIATED_BCR_REGULATION	2.162	0.001	0.017	0.015
REACTOME_FCGR_ACTIVATION	2.157	0.001	0.017	0.015
REACTOME_CREATION_OF_C4_AND_C2_ACTIVATORS	2.131	0.001	0.017	0.015
REACTOME_ANTIGEN_ACTIVATES_B_CELL_RECEPTOR_BCR	2.087	0.001	0.017	0.015
REACTOME_FCERI_MEDIATED_NF_kB_ACTIVATION	1.936	0.001	0.017	0.015
WP_SREBF_AND_MIR33_IN_CHOLESTEROL_AND_LIPID_HOMEOSTASIS	−1.907	−0.008	0.087	0.077
REACTOME_FATTY_ACIDS	−1.906	−0.006	0.073	0.065
REACTOME_ACTIVATION_OF_GENE_EXPRESSION_BY_SREBF_SREBP_	−1.870	−0.026	0.205	0.182
REACTOME_XENOBIOTICS	−1.825	−0.01	0.107	0.095
WP_CHOLESTEROL_BIOSYNTHESIS_PATHWAY	−1.778	−0.013	0.123	0.109
KEGG_TERPENOID_BACKBONE_BIOSYNTHESIS	−1.712	−0.013	0.123	0.109

CISD2: CDGSH iron sulfur domain 2; NOM: nominal; NES: normalized enrichment score; FDR: false discovery rate.

**Table 5:** Correlations between CISD2 and gene markers of immune cells in TIMER.

Cell type	Gene marker	GBM				LGG			
		None		Purity		None		Purity	
		Cor	*P* value	Cor	*P* value	Cor	*P* value	Cor	*P* value
B cells	CD19	−0.166	∗	−0.174	∗	0.006	0.893	0.018	0.696
	CD79A	0.089	0.275	0.095	0.267	−0.161	∗∗∗	-0.158	∗∗∗

T cells (general)	CD2	0.031	0.699	0.007	0.934	0.147	∗∗∗	0.158	∗∗∗
	CD3D	0.077	0.342	0.056	0.511	0.106	∗	0.115	∗
	CD3E	−0.038	0.637	−0.075	0.382	0.105	∗	0.110	∗

CD8+ T cells	CD8A	0.225	∗∗	0.202	∗	0.350	∗∗∗	0.353	∗∗∗
	CD8B	0.095	0.240	0.059	0.491	−0.010	0.816	−0.006	0.893

Monocyte	CD86	0.044	0.589	0.035	0.684	−0.090	∗	−0.089	0.050

TAM	CCL2	0.046	0.568	0.033	0.700	−0.046	0.297	−0.035	0.450
	CD68	0.033	0.684	0.019	0.824	−0.113	∗	−0.111	∗
	IL10	0.097	0.234	0.075	0.383	−0.010	0.819	0.014	0.759

M1	INOS (NOS2)	−0.155	0.056	−0.140	0.101	0.221	∗∗∗	0.225	∗∗∗
	IRF5	−0.166	∗	−0.200	∗	−0.129	∗∗	−0.131	∗∗

M2	CD163	0.122	0.132	0.124	0.146	0.021	0.629	0.028	0.543
	MS4A4A	0.210	∗∗	0.188	∗	−0.120	∗∗	−0.120	∗∗
	VSIG4	0.135	0.097	0.122	0.153	−0.170	∗∗∗	−0.175	∗∗∗

Neutrophils	CCR7	0.165	∗	0.184	∗	0.171	∗∗∗	0.189	∗∗∗
	CD66b (CEACAM8)	0.095	0.242	0.074	0.388	−0.081	0.066	−0.083	0.068
	CD11b (ITGAM)	−0.142	0.079	−0.140	0.102	−0.127	∗∗	−0.129	∗∗

NK cell	KIR2DL1	0.025	0.755	−0.009	0.913	−0.019	0.664	−0.035	0.441
	KIR2DL3	−0.049	0.548	−0.052	0.547	0.049	0.263	0.044	0.338
	KIR2DS4	−0.081	0.319	−0.112	0.190	0.035	0.429	0.048	0.290
	KIR3DL1	−0.052	0.521	−0.052	0.541	0.113	∗	0.107	∗
	KIR3DL2	−0.085	0.298	−0.094	0.271	0.013	0.774	0.004	0.922
	KIR3DL3	−0.072	0.375	−0.060	0.487	−0.036	0.410	−0.032	0.485

DC	BDCA-1 (CD1C)	0.110	0.178	0.123	0.150	−0.026	0.562	−0.020	0.661
	CD11c (ITGAX)	−0.161	∗	−0.159	0.062	−0.154	∗∗∗	−0.161	∗∗∗
	HLA-DPA1	0.012	0.887	−0.022	0.798	0.046	0.299	0.062	0.175
	HLA-DPB1	−0.012	0.880	−0.036	0.675	−0.010	0.817	0.003	0.955
	HLA-DQB1	0.018	0.822	0.022	0.795	0.019	0.661	0.029	0.530
	HLA-DRA	0.035	0.671	0.009	0.913	0.019	0.664	0.031	0.496

Th1	INF-*γ* (IFNG)	0.078	0.335	0.063	0.464	0.103	∗	0.111	∗
	STAT1	−0.155	0.056	−0.173	∗	0.369	∗∗∗	0.367	∗∗∗
	STAT4	0.037	0.649	0.010	0.908	0.363	∗∗∗	0.364	∗∗∗
	T-bet (TBX21)	0.080	0.328	0.087	0.308	0.101	∗	0.112	∗
	TNF*α* (TNF)	−0.048	0.558	−0.059	0.495	−0.149	∗∗∗	-0.148	∗∗

Th2	STAT5A	−0.331	∗∗∗	−0.352	∗∗∗	−0.096	∗	-0.092	∗
	STAT6	−0.113	0.164	−0.095	0.266	0.182	∗∗∗	0.174	∗∗∗
	GATA3	−0.152	0.060	−0.159	0.063	0.070	0.112	0.090	∗
	IL13	−0.046	0.571	−0.095	0.267	−0.036	0.412	−0.054	0.237

Tfh	BCL6	−0.134	0.100	−0.156	0.068	−0.205	∗∗∗	−0.199	∗∗∗
	IL21	0.047	0.562	0.052	0.546	0.054	0.220	0.057	0.214

Th17	IL17A	0.040	0.627	0.040	0.640	0.001	0.976	0.016	0.720

Treg	CCR8	0.004	0.963	−0.023	0.785	0.166	∗∗∗	0.167	∗∗∗
	FOXP3	−0.098	0.226	−0.150	0.078	0.321	∗∗∗	0.327	∗∗∗
	STAT5B	−0.214	∗∗	−0.225	∗∗	0.101	∗	0.111	∗
	TGF*β* (TGFB1)	−0.200	0.013	−0.181	0.034	−0.231	∗∗∗	−0.234	∗∗∗

T cell exhaustion	CTLA4	−0.057	0.486	−0.094	0.273	0.075	0.088	0.100	∗
	GZMB	−0.006	0.941	−0.015	0.864	0.134	∗∗	0.143	∗∗
	PD-1 (PDCD1)	0.114	0.161	0.094	0.275	0.045	0.306	0.032	0.483
	TIM3 (HAVCR2)	0.049	0.551	0.043	0.618	−0.094	∗	−0.095	∗

## Data Availability

The public datasets used in our work can be found on https://cance.rgenome.nih.gov/, http://www.linkedomics.org/login.php, https://www.proteinatlas.org/,http://cistrome.org/TIMER/, and http://gepia.cancer-pku.cn/index.html.

## Abbreviations

CISD2: CDGSH iron sulfur domain 2
*IDH*: Isocitrate dehydrogenase
TCGA: The Cancer Genome Atlas
GEO: Gene Expression Omnibus
FDR: False discovery rate
GSEA: Gene set enrichment analysis
GO: Gene Ontology
KEGG: Kyoto Encyclopedia of Genes and Genomes.

## Supplementary Materials

Supplementary Materials Supplementary Table 1: list of top 50 significant genes positively correlated with CISD2 expression in glioma. Supplementary Table 2: list of top 50 significant genes negatively correlated with CISD2 expression in glioma. Supplementary Table 3: GO and KEGG analyses of the top 5 significant pathways involved in glioma according to CISD2 expression.
