# Supplementary material for: High Expression of CISD2 in Relation to Adverse Outcome and Abnormal Immune Cell Infiltration in Glioma
Source: Dis Markers. 2022 Apr 21;2022:8133505. doi: 10.1155/2022/8133505 (PMC9050253; doi:10.1155/2022/8133505)
Supplement: Supplementary Materials — Supplementary Table 1: list of top 50 significant genes positively correlated with CISD2 expression in glioma. Supplementary Table 2: list of top 50 significant genes negatively correlated with CISD2 expression in glioma. Supplementary Table 3: GO and KEGG analyses of the top 5 significant pathways involved in glioma according to CISD2 expression. [file 8133505.f1.zip › Supplementary Table 1.docx]

TABLE S1: Top 50 significant genes positively correlated with CISD2 expression in glioma.

| Target molecule | Positively correlated molecule | Correlation coefficient (Pearson) | *P* value (Pearson) | Correlation coefficient (Spearman) | *P* value (Spearman) |
| --- | --- | --- | --- | --- | --- |
| CISD2 | COPS3 | 0.809 | <0.001 | 0.805 | <0.001 |
| CISD2 | EIF4E | 0.787 | <0.001 | 0.782 | <0.001 |
| CISD2 | ELOC | 0.786 | <0.001 | 0.763 | <0.001 |
| CISD2 | UBE2D3 | 0.782 | <0.001 | 0.759 | <0.001 |
| CISD2 | RAN | 0.782 | <0.001 | 0.759 | <0.001 |
| CISD2 | MMADHC | 0.770 | <0.001 | 0.752 | <0.001 |
| CISD2 | H2AZ1 | 0.769 | <0.001 | 0.733 | <0.001 |
| CISD2 | HCCS | 0.768 | <0.001 | 0.796 | <0.001 |
| CISD2 | CNIH4 | 0.760 | <0.001 | 0.736 | <0.001 |
| CISD2 | PGK1 | 0.760 | <0.001 | 0.780 | <0.001 |
| CISD2 | SLBP | 0.753 | <0.001 | 0.772 | <0.001 |
| CISD2 | TMEM70 | 0.750 | <0.001 | 0.769 | <0.001 |
| CISD2 | PSMD14 | 0.747 | <0.001 | 0.731 | <0.001 |
| CISD2 | OSTC | 0.744 | <0.001 | 0.718 | <0.001 |
| CISD2 | GLRX2 | 0.743 | <0.001 | 0.751 | <0.001 |
| CISD2 | GGH | 0.739 | <0.001 | 0.722 | <0.001 |
| CISD2 | AP3S1 | 0.736 | <0.001 | 0.754 | <0.001 |
| CISD2 | TXNDC9 | 0.733 | <0.001 | 0.711 | <0.001 |
| CISD2 | COMMD8 | 0.733 | <0.001 | 0.721 | <0.001 |
| CISD2 | TMED2 | 0.733 | <0.001 | 0.714 | <0.001 |
| CISD2 | BOLA3 | 0.731 | <0.001 | 0.720 | <0.001 |
| CISD2 | C1D | 0.730 | <0.001 | 0.713 | <0.001 |
| CISD2 | HINT1 | 0.730 | <0.001 | 0.675 | <0.001 |
| CISD2 | RPA3 | 0.729 | <0.001 | 0.715 | <0.001 |
| CISD2 | LYPLA1 | 0.729 | <0.001 | 0.713 | <0.001 |
| CISD2 | PARPBP | 0.728 | <0.001 | 0.706 | <0.001 |
| CISD2 | TIPRL | 0.728 | <0.001 | 0.720 | <0.001 |
| CISD2 | SEPHS2 | 0.728 | <0.001 | 0.753 | <0.001 |
| CISD2 | VPS29 | 0.726 | <0.001 | 0.707 | <0.001 |
| CISD2 | MED31 | 0.726 | <0.001 | 0.700 | <0.001 |
| CISD2 | GPN1 | 0.725 | <0.001 | 0.697 | <0.001 |
| CISD2 | PSMA1 | 0.725 | <0.001 | 0.705 | <0.001 |
| CISD2 | DCUN1D5 | 0.724 | <0.001 | 0.707 | <0.001 |
| CISD2 | MRPS10 | 0.723 | <0.001 | 0.709 | <0.001 |
| CISD2 | C3orf38 | 0.723 | <0.001 | 0.711 | <0.001 |
| CISD2 | ACP1 | 0.722 | <0.001 | 0.743 | <0.001 |
| CISD2 | SUMO1 | 0.722 | <0.001 | 0.694 | <0.001 |
| CISD2 | PNO1 | 0.719 | <0.001 | 0.712 | <0.001 |
| CISD2 | CCNB1 | 0.719 | <0.001 | 0.703 | <0.001 |
| CISD2 | TFB2M | 0.719 | <0.001 | 0.727 | <0.001 |
| CISD2 | RAB5IF | 0.717 | <0.001 | 0.683 | <0.001 |
| CISD2 | COPS4 | 0.717 | <0.001 | 0.694 | <0.001 |
| CISD2 | UBE2T | 0.717 | <0.001 | 0.711 | <0.001 |
| CISD2 | MAD2L1 | 0.716 | <0.001 | 0.679 | <0.001 |
| CISD2 | DPM1 | 0.715 | <0.001 | 0.700 | <0.001 |
| CISD2 | KPNA2 | 0.714 | <0.001 | 0.725 | <0.001 |
| CISD2 | YIPF5 | 0.714 | <0.001 | 0.738 | <0.001 |
| CISD2 | CKS1B | 0.714 | <0.001 | 0.691 | <0.001 |
| CISD2 | PPIA | 0.712 | <0.001 | 0.697 | <0.001 |
| CISD2 | ORC5 | 0.712 | <0.001 | 0.724 | <0.001 |
