# Supplementary material for: High Expression of CISD2 in Relation to Adverse Outcome and Abnormal Immune Cell Infiltration in Glioma
Source: Dis Markers. 2022 Apr 21;2022:8133505. doi: 10.1155/2022/8133505 (PMC9050253; doi:10.1155/2022/8133505)
Supplement: Supplementary Materials — Supplementary Table 1: list of top 50 significant genes positively correlated with CISD2 expression in glioma. Supplementary Table 2: list of top 50 significant genes negatively correlated with CISD2 expression in glioma. Supplementary Table 3: GO and KEGG analyses of the top 5 significant pathways involved in glioma according to CISD2 expression. [file 8133505.f1.zip › Supplementary Table 2.docx]

TABLE S2: Top 50 significant genes negatively correlated with CISD2 expression in glioma.

| Target molecule | Negatively correlated molecule | Correlation coefficient (Pearson) | *P* value (Pearson) | Correlation coefficient (Spearman) | *P* value (Spearman) |
| --- | --- | --- | --- | --- | --- |
| CISD2 | MT-ND4 | −0.690 | <0.001 | −0.695 | <0.001 |
| CISD2 | MT-CYB | −0.632 | <0.001 | −0.627 | <0.001 |
| CISD2 | RNU6-529P | −0.628 | <0.001 | −0.638 | <0.001 |
| CISD2 | MT-ND6 | −0.624 | <0.001 | −0.632 | <0.001 |
| CISD2 | MT-ATP6 | −0.623 | <0.001 | −0.606 | <0.001 |
| CISD2 | MT-CO2 | −0.623 | <0.001 | −0.594 | <0.001 |
| CISD2 | MT-ND5 | −0.605 | <0.001 | −0.614 | <0.001 |
| CISD2 | MT-ND3 | −0.603 | <0.001 | −0.596 | <0.001 |
| CISD2 | NET1 | −0.599 | <0.001 | −0.575 | <0.001 |
| CISD2 | FBXW4 | −0.591 | <0.001 | −0.579 | <0.001 |
| CISD2 | MT-RNR2 | −0.590 | <0.001 | −0.569 | <0.001 |
| CISD2 | HPSE2 | −0.581 | <0.001 | −0.612 | <0.001 |
| CISD2 | ADHFE1 | −0.577 | <0.001 | −0.577 | <0.001 |
| CISD2 | FLJ16779 | −0.573 | <0.001 | −0.591 | <0.001 |
| CISD2 | DEGS2 | −0.571 | <0.001 | −0.551 | <0.001 |
| CISD2 | ABLIM1 | −0.566 | <0.001 | −0.515 | <0.001 |
| CISD2 | LINC01561 | −0.565 | <0.001 | −0.586 | <0.001 |
| CISD2 | NDRG2 | −0.563 | <0.001 | −0.594 | <0.001 |
| CISD2 | MT-CO1 | −0.562 | <0.001 | −0.531 | <0.001 |
| CISD2 | FRA10AC1 | −0.559 | <0.001 | −0.515 | <0.001 |
| CISD2 | TAS2R4 | −0.557 | <0.001 | −0.550 | <0.001 |
| CISD2 | MT-CO3 | −0.554 | <0.001 | −0.523 | <0.001 |
| CISD2 | ZNNT1 | −0.552 | <0.001 | −0.535 | <0.001 |
| CISD2 | MT-ND2 | −0.548 | <0.001 | −0.513 | <0.001 |
| CISD2 | MT-TT | −0.545 | <0.001 | −0.482 | <0.001 |
| CISD2 | ZBTB47 | −0.544 | <0.001 | −0.496 | <0.001 |
| CISD2 | ALDH2 | −0.539 | <0.001 | −0.541 | <0.001 |
| CISD2 | MTSS2 | −0.539 | <0.001 | −0.522 | <0.001 |
| CISD2 | AC073896.4 | −0.538 | <0.001 | −0.515 | <0.001 |
| CISD2 | HMGN5 | −0.536 | <0.001 | −0.504 | <0.001 |
| CISD2 | IGIP | −0.535 | <0.001 | −0.468 | <0.001 |
| CISD2 | FAM171A1 | −0.530 | <0.001 | −0.473 | <0.001 |
| CISD2 | HIPK2 | −0.528 | <0.001 | −0.512 | <0.001 |
| CISD2 | AC015540.1 | −0.528 | <0.001 | −0.528 | <0.001 |
| CISD2 | SLC2A4 | −0.527 | <0.001 | −0.561 | <0.001 |
| CISD2 | SLC25A21-AS1 | −0.525 | <0.001 | −0.481 | <0.001 |
| CISD2 | AL157700.1 | −0.525 | <0.001 | −0.526 | <0.001 |
| CISD2 | AC055720.2 | −0.524 | <0.001 | −0.548 | <0.001 |
| CISD2 | LINC02636 | −0.523 | <0.001 | −0.538 | <0.001 |
| CISD2 | ZNF710-AS1 | −0.523 | <0.001 | −0.501 | <0.001 |
| CISD2 | AC093535.2 | −0.523 | <0.001 | −0.538 | <0.001 |
| CISD2 | LINC00634 | −0.521 | <0.001 | −0.521 | <0.001 |
| CISD2 | MTATP6P1 | −0.520 | <0.001 | −0.589 | <0.001 |
| CISD2 | PC | −0.520 | <0.001 | −0.495 | <0.001 |
| CISD2 | MT-ND1 | −0.520 | <0.001 | −0.480 | <0.001 |
| CISD2 | MT-RNR1 | −0.517 | <0.001 | −0.516 | <0.001 |
| CISD2 | CDH20 | −0.516 | <0.001 | −0.526 | <0.001 |
| CISD2 | SLCO1A2 | −0.516 | <0.001 | −0.504 | <0.001 |
| CISD2 | MIR9-3HG | −0.514 | <0.001 | −0.507 | <0.001 |
| CISD2 | AL157392.3 | −0.512 | <0.001 | −0.464 | <0.001 |
