# Supplementary material for: High Expression of CISD2 in Relation to Adverse Outcome and Abnormal Immune Cell Infiltration in Glioma
Source: Dis Markers. 2022 Apr 21;2022:8133505. doi: 10.1155/2022/8133505 (PMC9050253; doi:10.1155/2022/8133505)
Supplement: Supplementary Materials — Supplementary Table 1: list of top 50 significant genes positively correlated with CISD2 expression in glioma. Supplementary Table 2: list of top 50 significant genes negatively correlated with CISD2 expression in glioma. Supplementary Table 3: GO and KEGG analyses of the top 5 significant pathways involved in glioma according to CISD2 expression. [file 8133505.f1.zip › Supplementary Table 3.docx]

TABLE S3: GO and KEGG pathway analyses of the top five significant pathways involved in glioma according to CISD2 expression level.

| Ontology | ID | Description | GeneRatio | BgRatio | *p-*value | *p*.adjust | q-value |
| --- | --- | --- | --- | --- | --- | --- | --- |
| BP | GO:0043312 | neutrophil degranulation | 412/12755 | 485/18670 | 1.46e-17 | 4.26e-14 | 3.13e-14 |
| BP | GO:0042119 | neutrophil activation | 422/12755 | 498/18670 | 1.48e-17 | 4.26e-14 | 3.13e-14 |
| BP | GO:0002283 | neutrophil activation involved in immune response | 414/12755 | 488/18670 | 1.98e-17 | 4.26e-14 | 3.13e-14 |
| BP | GO:0002446 | neutrophil mediated immunity | 422/12755 | 499/18670 | 3.10e-17 | 5.01e-14 | 3.68e-14 |
| BP | GO:1903706 | regulation of hemopoiesis | 403/12755 | 475/18670 | 5.19e-17 | 6.71e-14 | 4.93e-14 |
| CC | GO:0005743 | mitochondrial inner membrane | 396/13378 | 473/19717 | 1.73e-15 | 1.35e-12 | 8.98e-13 |
| CC | GO:0098798 | mitochondrial protein complex | 228/13378 | 262/19717 | 4.45e-13 | 1.74e-10 | 1.16e-10 |
| CC | GO:0031983 | vesicle lumen | 286/13378 | 339/19717 | 2.28e-12 | 4.46e-10 | 2.96e-10 |
| CC | GO:0034774 | secretory granule lumen | 272/13378 | 321/19717 | 2.77e-12 | 4.46e-10 | 2.96e-10 |
| CC | GO:0060205 | cytoplasmic vesicle lumen | 285/13378 | 338/19717 | 2.86e-12 | 4.46e-10 | 2.96e-10 |
| MF | GO:0044389 | ubiquitin-like protein ligase binding | 253/12504 | 308/17697 | 2.08e-06 | 0.002 | 0.002 |
| MF | GO:0031625 | ubiquitin protein ligase binding | 238/12504 | 290/17697 | 4.74e-06 | 0.003 | 0.003 |
| MF | GO:0050839 | cell adhesion molecule binding | 395/12504 | 499/17697 | 8.16e-06 | 0.003 | 0.003 |
| MF | GO:0019902 | phosphatase binding | 156/12504 | 185/17697 | 1.06e-05 | 0.003 | 0.003 |
| MF | GO:0003697 | single-stranded DNA binding | 99/12504 | 113/17697 | 1.58e-05 | 0.004 | 0.003 |
| KEGG | hsa05132 | Salmonella infection | 210/5680 | 249/8076 | 1.51e-07 | 3.81e-05 | 2.70e-05 |
| KEGG | hsa04110 | Cell cycle | 111/5680 | 124/8076 | 2.35e-07 | 3.81e-05 | 2.70e-05 |
| KEGG | hsa05131 | Shigellosis | 205/5680 | 246/8076 | 1.35e-06 | 1.46e-04 | 1.03e-04 |
| KEGG | hsa03050 | Proteasome | 45/5680 | 46/8076 | 1.81e-06 | 1.47e-04 | 1.04e-04 |
| KEGG | hsa05203 | Viral carcinogenesis | 171/5680 | 204/8076 | 4.91e-06 | 3.18e-04 | 2.25e-04 |

CISD2: CDGSH iron sulfur domain 2; BP: biological process; CC: cellular component; MF: molecular function.
